# Diagnosis of jejunal tubular duplication by enteroscopy in an adult female with recurrent gastrointestinal bleeding

**DOI:** 10.1055/a-2748-1177

**Published:** 2025-12-15

**Authors:** Wentong Lan, Meimei Hu, Yinglin Liao, Kaitao Yuan, Ning Zhang, Danping Zheng

**Affiliations:** 1 71068 Department of Endoscopy, The First Affiliated Hospital, Sun Yat-sen University, Guangzhou, China; 2 71068 Department of General Surgery, The First Affiliated Hospital, Sun Yat-sen University, Guangzhou, China; 3 71068 Department of Gastroenterology, The First Affiliated Hospital, Sun Yat-sen University, Guangzhou, China

Intestinal duplication, uncommon in adulthood, is a rare congenital deformity that mainly occurs in the ileum 1 . It can be divided into two types: cyst and tubular. The diagnosis of intestinal duplication is challenging due to its atypical symptoms and limitations of current imaging techniques 2 .

A 57-year-old woman presented with recurrent melena for 1 month. Routine blood tests revealed a decreased hemoglobin level of 73g/L (reference range: 115–150 g/L). Neither upper endoscopy nor colonoscopy revealed obvious findings, suggesting suspected small bowel bleeding. An abdominal computed tomography enterography demonstrated no evident abnormality. Capsule endoscopy showed a protruding lesion with active bleeding in the upper jejunum ( Fig. 1 ). A subsequent transoral double-balloon enteroscopy was performed, achieving a complete, unidirectional examination of the entire small bowel.

**Fig. 1:**
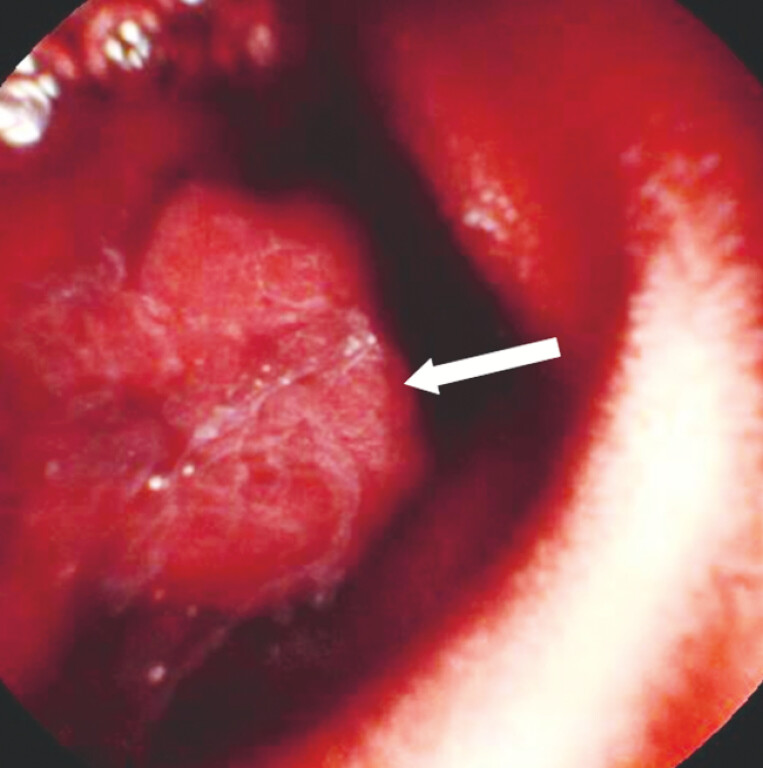
A capsule endoscopic image showing a protruding lesion (white arrow) with active bleeding in the proximal jejunum.

We spotted a triple-lumen appearance along with exposed swollen blood vessels ( Fig. 2 ) at the proximal part of jejunum. Closer observation of the triple-lumen structure found a diverticular opening and two separate, distinct lumina ( Fig. 3 ) within the small intestine, both open to the distal end of the jejunum ( Video 1 ), suggesting intestinal tubular duplication. No abnormalities were detected in the rest of the jejunum and the ileum. Partial surgical resection of the small intestine was performed for the patient, revealing a 3 cm × 2 cm tubular structure resembling a smaller intestine attached to the bowel wall of the main jejunum ( Fig. 4 ). Pathology of the surgical specimen exhibited the presence of intestinal mucosa and submucosa inside the tubular lesion, confirming the the diagnosis of intestinal duplication.

**Fig. 2:**
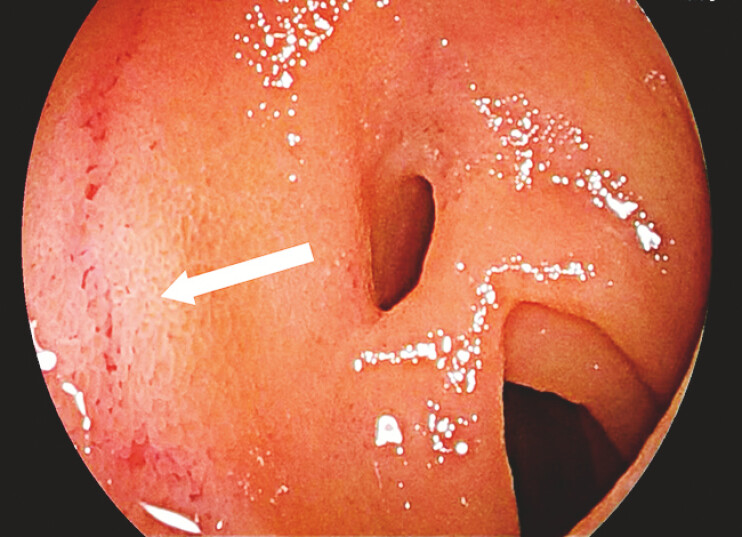
An endoscopic image showing exposed swollen blood vessels (white arrow) near the triple-lumen structure.

**Fig. 3:**
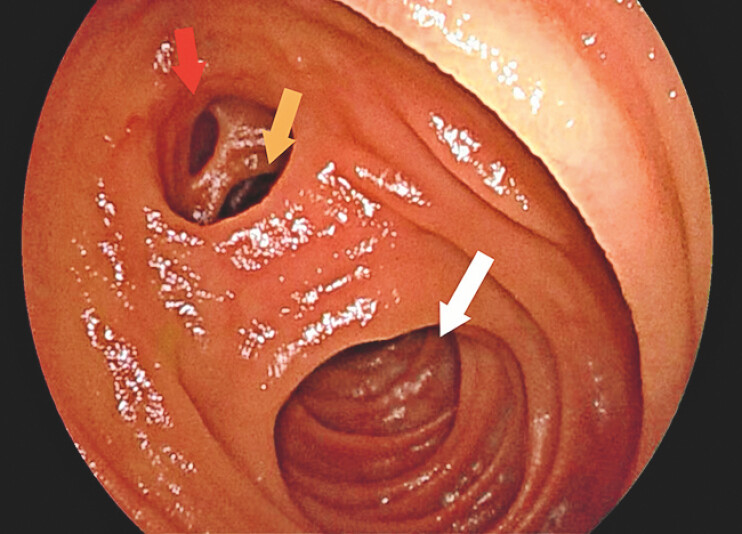
An endoscopic image showing the triple-lumen structure including the diverticular opening (red arrow), the duplicate lumen (yellow arrow) and the main lumen (white arrow).

Diagnosis of small bowel tubular duplication with enteroscopy.Video 1

**Fig. 4:**
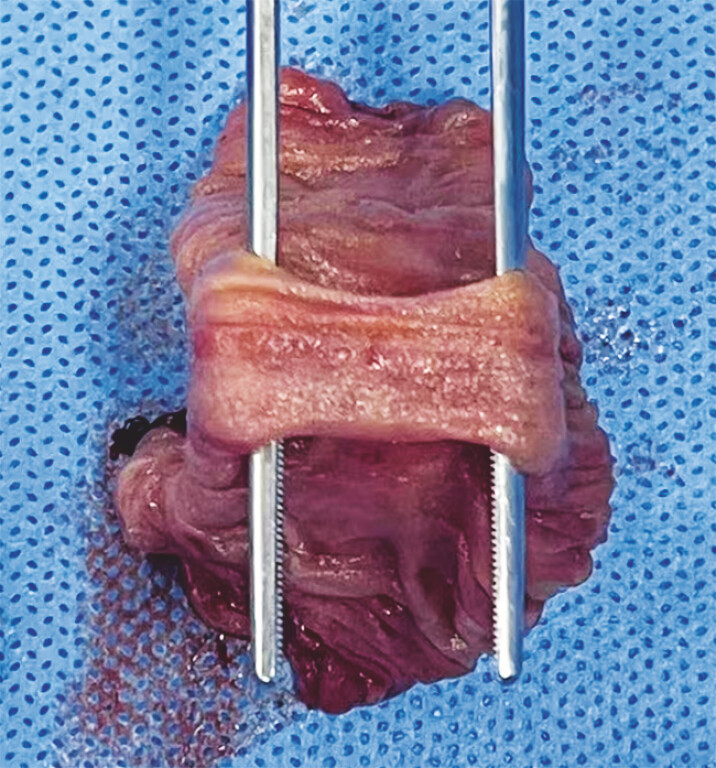
A surgical specimen showing a 3 × 2 cm tubular structure attached to the bowel wall of the jejunum.

In this case, we successfully diagnosed a jejunal tubular duplication causing gastrointestinal bleeding in an adult using trans-oral enteroscopy. This highlights the significance of enteroscopy in identifying rare and complex small intestinal lesions with atypical symptoms, especially when regular imaging studies or capsule endoscopy fail to make a diagnosis.

Endoscopy_UCTN_Code_CCL_1AC_2AF

## Notes

Endoscopy E-Videos https://eref.thieme.de/e-videos
*E-Videos* is an open access online section of the journal *Endoscopy* , reporting on interesting cases and new techniques in gastroenterological endoscopy. All papers include a high-quality video and are published with a Creative Commons CC-BY license. Endoscopy E-Videos qualify for HINARI discounts and waivers and eligibility is automatically checked during the submission process. We grant 100% waivers to articles whose corresponding authors are based in Group A countries and 50% waivers to those who are based in Group B countries as classified by Research4Life (see: https://www.research4life.org/access/eligibility/ ). This section has its own submission website at https://mc.manuscriptcentral.com/e-videos .
